# LC-MS-Based Lipidomic Analysis of Serum Samples from Patients with Type 2 Diabetes Mellitus (T2DM)

**DOI:** 10.1155/2022/5559470

**Published:** 2022-02-12

**Authors:** Jia Liu, Lu Bai, Weimin Wang, Yuqing Song, Wenbo Zhao, Qingwei Li, Qiming Wu

**Affiliations:** ^1^Institute of Systems Biomedicine, Department of Pathology, School of Basic Medical Sciences, Beijing Key Laboratory of Tumor Systems Biology, Peking-Tsinghua Center for Life Sciences, Peking University Health Science Center, Beijing 100191, China; ^2^The First Affiliated Hospital of Hebei North University, Zhangjiakou, Hebei 075000, China; ^3^Department of Cardiology, Beijing Ditan Hospital, Capital Medical University, Beijing 100015, China; ^4^Department of Cardiology, Peking University People's Hospital, Beijing 100044, China; ^5^State Key Laboratory of NBC Protection for Civilian, Beijing 102205, China

## Abstract

**Background:**

With the development of social economy, type 2 diabetes mellitus (T2DM) is becoming a severe health problem globally.

**Methods:**

To systematically understand the lipid metabolism in T2DM, we applied untargeted lipidomics to the serum of T2DM patients and control group using ultrahigh-performance liquid chromatography (UHPLC) coupled with high-resolution mass spectrometry (MS).

**Results:**

Over two thousand molecular features were detected by our approach, of which 222 lipid species in positive ion mode and 145 species in negative were reliably identified based on precise molecular weights and MS/MS patterns. Multivariate analysis was adopted to differentiate T2DM patients and the control group using principal component analysis (PCA) and orthogonal partial least squares discrimination analysis (OPLS-DA). The dysregulated lipid species were found and their significance in pathophysiology was discussed. Correlation analysis of selected lipids and important clinical variables was performed and addressed.

**Conclusions:**

This study unveils several new lipids and pathways considerably involved in T2DM and provides novel insights into understanding the pathogenesis underlying T2DM.

## 1. Introduction

With the development of social economy, type 2 diabetes mellitus (T2DM) is increasingly prevalent in the 21st century. The global prevalence of diabetes is estimated to be 9.3% (463 million people) in 2019 and likely to rise to 10.2% (578 million people) by 2030 [1]. Globally, T2DM is the leading cause of morbidity and mortality [2, 3]. The past 30 years have witnessed the sharply increased prevalence of diabetes in China [4]. In addition, studies indicated that metabolic syndrome (MetS) was also increased dramatically, and the proportion of adult people suffering metabolic syndrome was as high as 25 percent all over the world [5]. The global public health concern is not only the single factor about obesity, ageing, diet, and physical health but also humans. Metabolic changes affect body's internal balance and lead to severe complications in patients with T2DM. Thus, the identification of reliable metabolic changes of this severe disease helps reduce the disability rate of T2DM.

Metabolomics is an emerging technology that enables the global assessment of metabolites and their biological significance in diseases. There have been several recent metabolomic studies on T2DM [6–8], focus on the pathogenesis of this disease, the biomarkers associated and prediction of insulin resistance (IR), *etc*. Lipidomics, an important branch of metabolomics, aims to detect, quantify, and pinpoint all lipid species in a biological system [9, 10]. Ceramides were reported to be associated with a higher risk of diabetes and insulin resistant [11]. Free fatty acids (FFA) could lead to insulin resistance by inhibiting the activity of PI3K. Phosphatidylethanolamine and acylcarnitines were also involved in high blood glucose and T2DM. With the advances of mass spectrometers and the software for qualitative and quantitative analysis, liquid chromatography-mass spectrometry- (LC-MS-) based lipidomics is increasingly applied to clinical and fundamental researches [12–14]. Several studies recently investigated lipid predictors and lipid pathways in humans with T2DM using the lipidomics approach [15, 16]. For instance, Lu et al. investigated lipidome changes in lipid coregulation antecedent to the T2DM using QTRAP 6500 PLUS with MRM scan mode [15, 16]. Lappas and co-workers identified lipids and lipid profiles that were predictive of the development of type 2 diabetes in women with a gestational diabetes mellitus (GDM) pregnancy also using 4000 Q/TRAP MRM scan mode [17]. However, the detection technology they adopted is targeted lipidomics or shotgun lipidomics rather than untargeted lipidomics. It is difficult to identify new lipid markers due to the technology's low resolution and low mass accuracy.

In this study, an untargeted lipidomics approach using ultrahigh-performance liquid chromatography coupled with an advanced high-resolution mass spectrometer Q-Exactive (UHPLC-Q-E-MS) was developed to profile lipid alterations in T2DM patients. Lipids could be reliably chemically attributed by their accurate *m/z* values and MS/MS spectra. The T2DM group and healthy controls were clearly distinguished by multivariate data analysis such as orthogonal partial least squares discrimination analysis discrimination analysis (OPLS-DA). Lipids were identified with accurate *m/z* values and their MS/MS spectrum patterns. This strategy may be helpful for the understanding and elucidation of the molecular mechanism of T2DM.

## 2. Experimental

### 2.1. Study Subjects and Inclusion Criteria

A total of 40 T2DM patients, diagnosed by World Health Organization criteria between April 2014 and December 2014, were recruited from the outpatient or hospitalized with diabetes in Peking University People's Hospital (Beijing, China). The inclusion criteria were age between 18 and 80 years old, body mass index between 20 and 35 kg/m^2^, and not having diet programs. The participants with severe active infectious diseases, any musculoskeletal diseases, malignant tumors, chronic inflammatory diseases, severe kidney failure (eGFR ≤ 25 ml/min × 1.73 mm^2^), severe heart failure (LVEF ≤ 30%), special diet, or recent weight changes were excluded. Patients with severe organ failure or severe chronic wasting disease that affects lipid metabolism were excluded, because they were in an environment of severe or extreme metabolic disorders. 47 healthy controls were age, gender, body mass index (BMI), and homocysteine (HCY) matched with the T2DM patients (Table 1). Blood samples were collected in procoagulation tube, and serum was recovered by centrifugation.

This study was approved by the Ethics Committee of the Peking University People's Hospital, and informed consents were obtained from all participants. All methods were performed in accordance with the relevant guidelines and regulations.

### 2.2. Sample Processing

Lipids in serum were extracted by a modified Folch method [18, 19]. Specifically, 267 *μ*L CHCl_3_ and 133 *μ*L methanol were added into 100 *μ*L samples. After sufficient vortexing and adequate centrifugation, the lower phase was recovered and evaporated by lyophilization. The resultant powder was resuspended in CHCl_3_/methanol for LC-MS/MS lipidomic analysis.

### 2.3. LC-MS Analysis

UHPLC-Q-Exactive MS (Thermo Scientific) using a reversed-phase C18 column (Xselect CSH) was employed for lipid analysis. The analytical column used was in line with other publication [20]. Detailed LC instrument conditions are listed in the supporting material in Supplementary Methods and Table S1.

For MS analysis, the source voltage was 3.3 kV for + ion mode and 2.8 kV for − ion mode. Quality control samples (QCs) were prepared by pooling real samples together and injected every ten runs during the whole sequence.

### 2.4. Data Treatment and Analysis

The raw data were first processed using MSDIAL for peak integration, retention time alignment, and chemical identification [21]. Statistical significance was calculated using the *t*-test, and the threshold of *p* value was 0.05. The retention time and intensities of the molecular features detected were uploaded into MetaboAnalyst [22] for multivariate analysis such as principal component analysis (PCA), heat map, and OPLS-DA.

## 3. Results

### 3.1. Clinical Characteristic of Subjects

A total of 40 T2DM patients and 47 healthy controls were enrolled in this open-label study. The clinical information of the subjects recruited is listed in Table 1. T2DM patients and healthy controls (HCs) were matched in terms of age, sex, body mass index (BMI), and homocysteine (HCY). HCY is primarily disposed via two methionine-conserving pathways and is an indicator of dietary habits [23]. HCY was matched between the two groups, reflecting the balanced diet of omnivorous Chinese people. As compared to healthy controls, T2DM patients had significantly higher fasting glucose, HbA1c, and lower high-density apolipoprotein (HDL).

### 3.2. Serum Lipidomic Profiles

**Figure 1:**
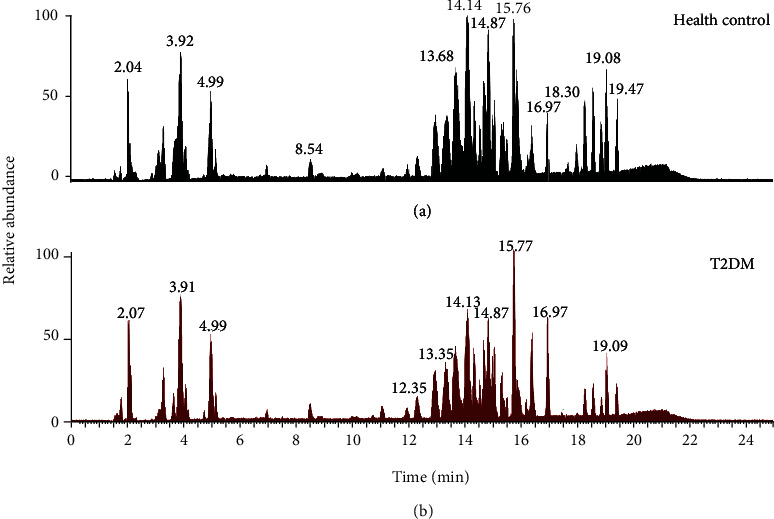
Full scan base peak mass chromatograms in positive mode: (a) healthy controls and (b) T2DM group.

**Figure 2:**
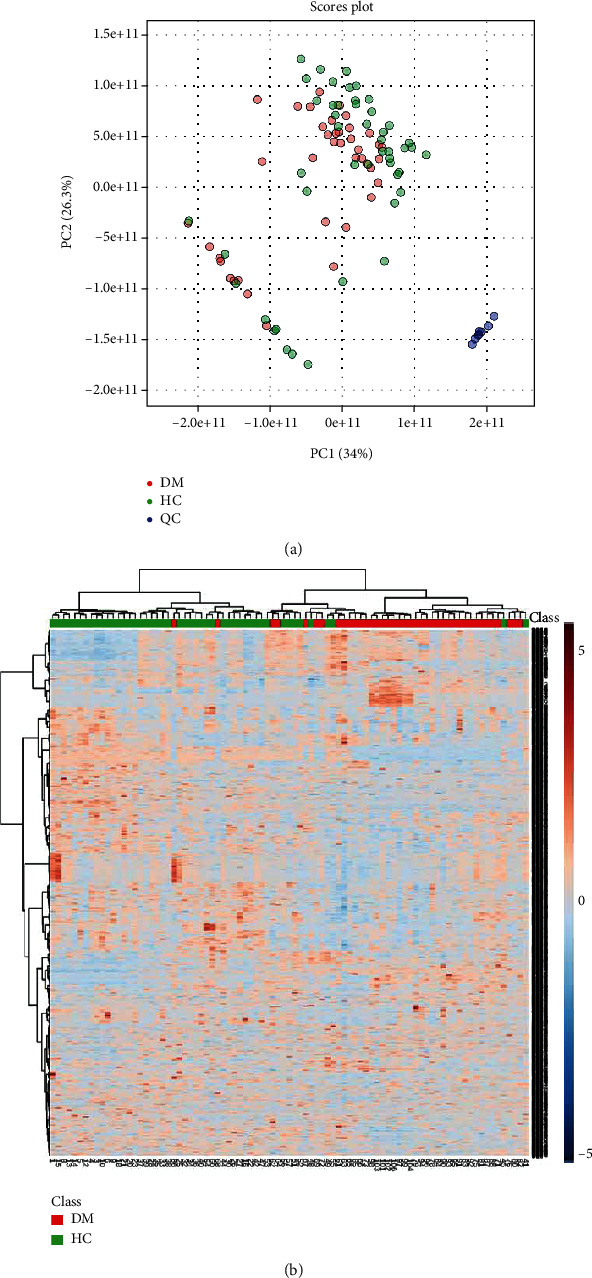
(a) PCA score plot of positive ion mode. (b) Clustering heat map by Pearson correlation of the samples. The rows and columns represent metabolites and sample hierarchical clustering, respectively. For group name, red reflects DM and green represents healthy controls. For the lipid expression, red indicates upregulation and blue reflects downregulation.

Samples were analyzed with UHPLC-MS in a data-dependent scan mode, and a number of lipid classes and subclasses was detected, including free fatty acids (FFA), phosphatidylcholine (PC), lysophosphatidylcholine (LPC), phosphatidylethanolamine (PE), lysophosphatidylethanolamine (LPE), phosphatidylserine (PS), phosphatidylinositol (PI), sphingomyelin (SM), monoglyceride (MG), and triacylglycerol (TG). Typical base peak ion chromatograms of the healthy controls (Figure 1(a)) and T2DM group (Figure 1(b)) in positive ion mode are shown. The analytical performance of the LC-MS-based lipidomics method was evaluated using pooled QC samples. All samples were run in a single sequence and QC samples were inserted every 10 sample injections. The QC samples were gathered together in PCA score plot, as shown in Figure 2(a). The retention time variation was within 0.2 minute (see Figure S1 in supporting material). These data reflected stability and reliability of our serum lipidomics instrument method.

### 3.3. Multivariate Analysis

**Figure 3:**
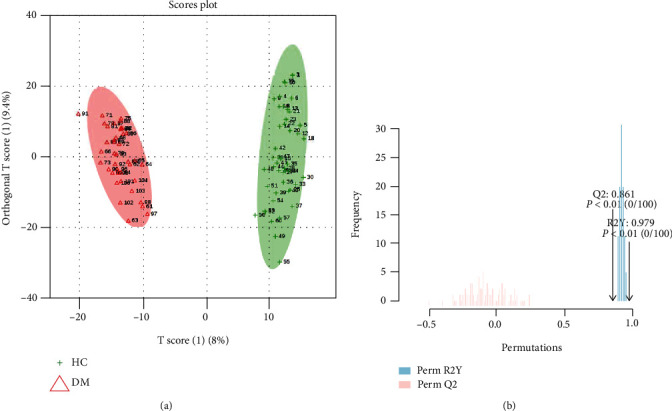
(a) OPLS-DA score plots based on metabolomics data. (b) Validation for over-fitting by permutation test. The predicted residual sum of squares Q2 (cum) was 0.861, and the fraction of the sum of squares R2Y was 0.979. The *p* value < 0.01 is at 100 permutations. These results indicated good predictive performance and there was no over-fitting in the model.

Raw data were processed on MS-DIAL software for peak integration, retention time alignment, and chemical identification. The *m/z* values, retention times, and peak abundances were extracted and then exploited for multivariate analysis. The PCA score plot containing the T2DM, HC, and QC groups in positive ion mode is shown in Figure 2(a).  Figure 2(b) illustrates the heat map via Pearson correlation, and the rows and columns represent metabolites and sample hierarchical clustering, respectively. Generally, samples were clustered in consistent with health status, which reflected a profound disturbance of lipid metabolism in these two statuses. To further differentiate the T2DM and HC groups and find potential biomarkers, we performed OPLS-DA, which is a supervised statistical algorithm, to classify and differentiate the T2DM and HC groups. In the OPLA-DA score plot (Figure 3(a)), a sound clustering tendency was observed between T2DM and HC groups. The over-fitting was validated by permutation testing, as shown in Figure 3(b). The predicted residual sum of squares Q2 (cum) was 0.861, and the fraction of the sum of squares R2Y was 0.979. The *p* value < 0.01 is at 100 permutations. These results indicated good predictive performance and there was no over-fitting in the model. Thus, substantial metabolic differences existed in the T2DM patient compared to healthy people.

### 3.4. Significantly Changed Lipids

**Figure 4:**
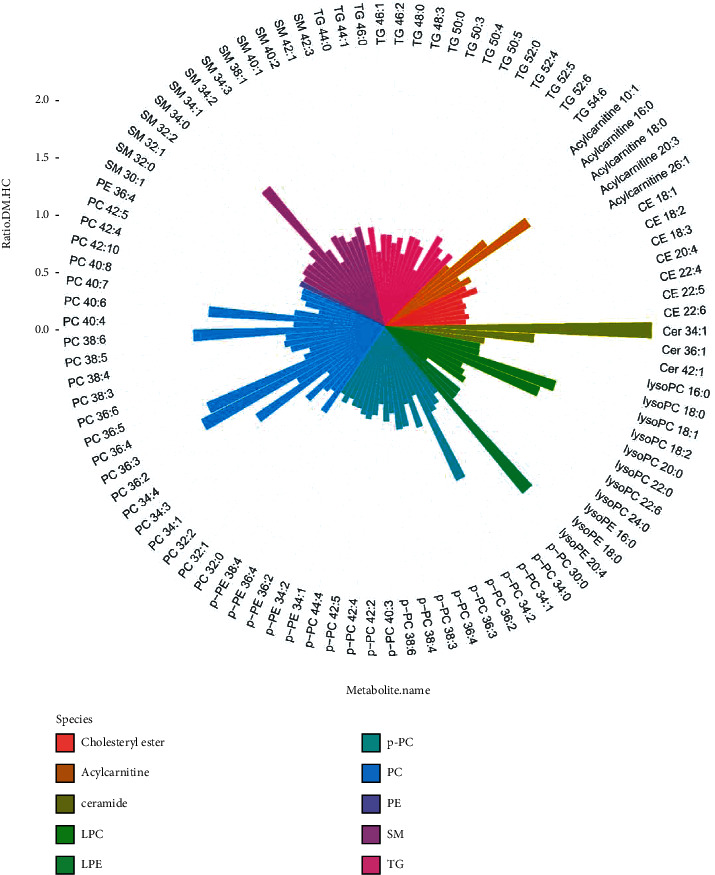
Bar plot in a polar coordinates showing the trend and fold change of lipids differentially expressed between DM and healthy controls. Each lipid class was assigned to a unique color. The length of each bar represents the fold change of each lipid.

The detected lipids were chemically attributed using MS-DIAL software by matching the measured MS and MS^2^ spectra with the lipidmap database. In total, 222 lipids in positive ion mode and 145 lipids in negative were identified, which belonged to 15 lipid classes. Among them, the most significantly dysregulated lipids are listed in Table 2 with both *p* value < 0.05 and fold change > 2 or < 0.5. Cholesteryl esters, PC, LPC, PE, plasmenyl-PC, SM, and TG were downregulated in T2DM patients with *p* value < 0.05. For other lipid classes, some species were upregulated and others were downregulated in T2DM patients. The trend and fold change of each lipid was illustrated in Figure 4, in which each lipid class was assigned to a unique color.

**Figure 5:**
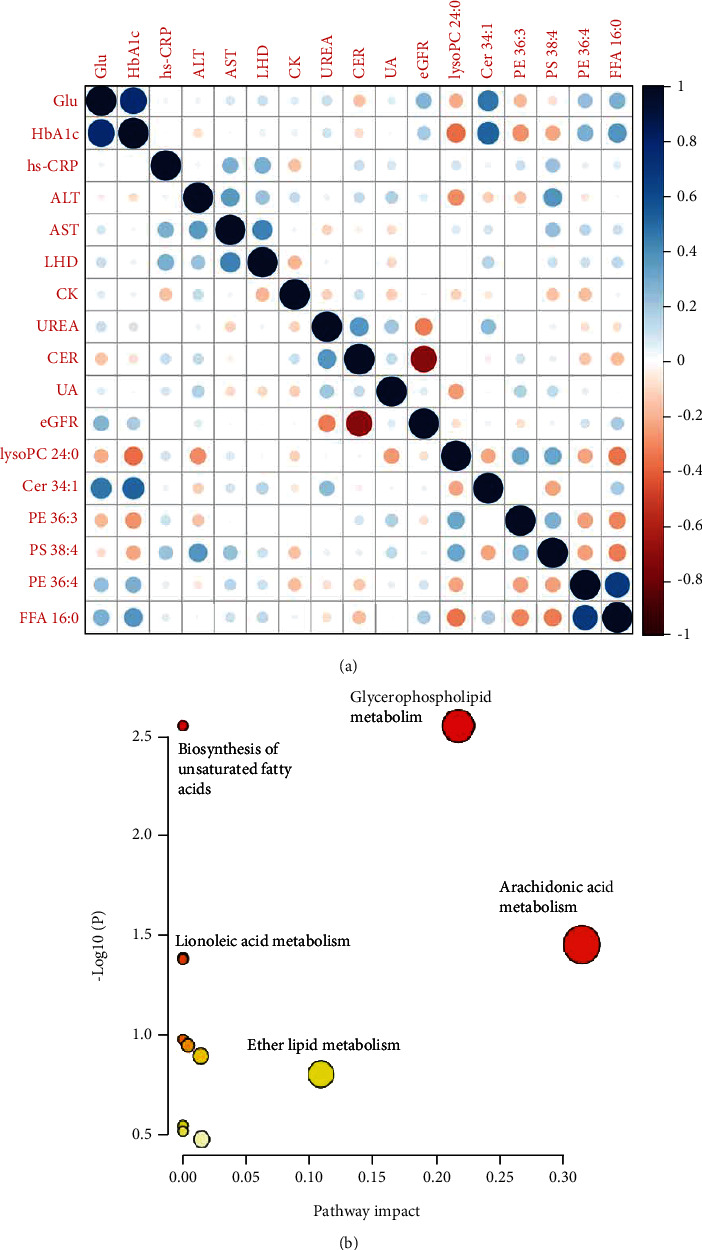
(a) Correlation analysis of key clinical status and the significantly changed lipids. The blue and red dots represent positive and negative correlation, respectively, and the dots size indicates the degree of correlation between these variables. (b) Pathway analysis indicated that several pathways involving the metabolism of glycerophospholipid and arachidonic acid were perturbed in T2DM patients compared with healthy controls.

To pinpoint the pathways that are dysregulated in T2DM patients compared to HC, the identified and significantly changed lipids detected in both positive and negative ion mode were input for KEGG-based pathway analysis. As illustrated in Figure 5(b), the pathways including the metabolism of glycerophospholipid and arachidonic acid were perturbed in T2DM patients compared with healthy controls. The relevance among important clinical variables and selected lipid species was revealed by correlation analysis in Figure 5(a). As expected, HbA1c was highly correlated with fasting glucose, and CER was negatively correlated with eGFR. Interestingly, ceramide 34 : 1 was also strongly negative relevant to fasting glucose and HbA1c, with correlation coefficient 0.47 and 0.52, respectively.

## 4. Discussion

Lipidomic profiling is a useful and powerful method in a variety of diseases, and it has been progressively employed in finding potential biomarkers, discovering therapeutic targets, and revealing traditional Chinese medicine mechanism [9, 10, 24]. T2DM, as a common metabolic disorder, is mainly characterized by hyperglycemia and insulin resistance (IR). In our study, the observed changes in lipid metabolism in serum are summarized in Table 2 and Figure 4. Moreover, our results uncovered the possible association between the traditional risk factors and key lipid species, as shown in Figure 5. We also try to find out and ascertain the dysregulation of lipid metabolism in serum of diabetic patients and to further clarify the potential biological mechanisms underlying the risk of diabetes.

Genetic studies demonstrated that human subjects in insulin resistant showed abnormal accumulation of ceramide [25]. Other clinical studies on American Indians indicated that higher concentrations of Cer-18, Cer-20, and Cer-22 were associated with a higher risk of diabetes [11]. Similarly, in our study, one of the most significant differences in serum lipidome between healthy controls and T2DM patients was also ceramide. Ceramide is a central molecule and a major second messenger in the sphingomyelin signaling pathway. It was reported that ceramide-rich low-density lipoprotein (LDL) had been shown to cause increased proinflammatory status of macrophages and insulin resistance in skeletal muscle aggregation [26]. It is believed that interleukin 6 (IL-6) played a part in the metabolic link among ceramides, inflammation, and IR [27]. Although the mechanistic basis of observed elevated serum ceramide levels in T2DM has not been fully established, ceramide is considered to be a proinflammatory lipid. A study showed that obese mice with hypoxia induce insulin resistance through the HIF-2*α*-NEU3-ceramide pathway [28]. In the current study, the concentration of Cer 34 : 1 was higher in T2DM and positively correlated to fasting glucose and HbA1c, reflecting the development of type 2 diabetes. We also found that there is a positive correlation between FFA 16 : 0 and ceramide 34 : 1, indicating that the saturated fatty acids and ceramide have potential biological activity against insulin resistance in diabetic patients.

Recently, several reviews considered the association between T2DM risk and lipid classes [29–31]. Free fatty acids (FFA) are the basic nutrients of the human body and play an essential role in human health. They could induce endoplasmic reticulum (ER) stress in pancreatic beta cells, which promotes inflammation, secretory dysfunction, and apoptosis [32, 33]. Shetty and Kumari summarized that FFA could lead to insulin resistance by inhibiting the activity of PI3K [34]. A consistent finding was reported that fatty acids associated with de novo lipogenesis (DNL), especially 16-carbon fatty acids, were significantly related to the incidence of T2DM [35]. Polyunsaturated fatty acids (PUFA), such as arachidonic acid (FFA 20 : 4), are composed of more than two double bonds. Arachidonic acid is one of the most important components of cell membrane, and it could protect RIN-5F (pancreatic *β*-cell) cells in T2DM from streptozotocin-induced cytotoxicity [36]. Endogenous arachidonic acid is mainly produced by cell membrane phospholipids catalyzed by enzymes of the phospholipase A2 (PLA2) superfamily [37]. Seyfarth et al. reported that the increase in average glucose concentration of T2DM adolescents was related to the decrease of lipoprotein-associated phospholipase A2 (Lp-PLA2) activity [38]. In our study, we found that arachidonic acid was negatively associated with T2DM, which was consistent with their work. In this way, FFA disrupts insulin signaling through toll-like receptor 4 (TLR4) pathway, resulting in insulin resistance (IR) [39, 40]. Pathway analysis also showed that arachidonic acid metabolism was dysregulated in T2DM (Figure 5). Palmitic acid (FFA16 : 0) may trigger the expression of interleukin-1 *β* and interleukin-6 in human coronary artery endothelial cells [41, 42]. In our study, we found that FFA 16 : 0 was upregulated in the T2DM group. Moreover, FFA 16 : 0 was positively associated with fasting glucose and HbA1c. Fatty acid with shorter chain and saturated CC bonds may stimulate the triggering development of T2DM. Conversely, fatty acids containing longer chain and unsaturated double bonds play protective function.

PE is an utmost structural lipid in the membrane, even it is a minor species in plasma. PE is synthesized via de novo and salvage pathways [43], and it is converted by ER-derived phosphatidylserine (PS) via decarboxylase phosphatidylserine decarboxylase 1 (Psd1) [44]. Previous studies have shown that PE was associated with high fasting blood glucose and T2DM [45, 46]. We reported that PE 36 : 4 was increased in T2DM compared with the healthy group and tended to be positively related to fasting glucose and HbA1c, which confirmed their studies. However, on the contrary, PE 36 : 3 was decreased in T2DM patients and inversely related to fasting glucose and HbA1c. This may be attributed to the different synthesis paths of these PEs. Decreased abundance of plasmalogen in the T2DM group indicated an increased in oxidative imbalance possibly caused by systemic inflammatory response. As an endogenous antioxidant medium for kinetics and membrane structure, plasmalogens could protect lipoproteins and membranes from oxidation [47, 48]. In our study, we also found that 5 species of plasmenyl-PE were decrease in T2DM, indicating the reduced antioxidant capacity in T2DM patients. Interestingly, our study reported that there was a strong positive correlation between PE 36 : 4 and FFA 16 : 0, although the mechanism underlying was not fully understood.

Acylcarnitines (ACCs) are the catabolic end products of fatty acids and several branched-chain amino acids that are utilized to generate cellular energy [49]. ACCs could be produced by shifting the activated fatty acids to carnitines at mitochondrial membrane [50]. A number of studies have reported an association between the accumulation of acylcarnitines and IR [51–53]. The mechanism involved may be that high level of glucose activates carnitine palmitoyltransferase I (CPT1) through oxidative stress. In detail, a slight increase in the rate of *β*-oxidation could lead to incomplete oxidation of fat, impaired conversion to carbohydrate oxidation, partial depletion of tricarboxylic acid (TCA) cycle intermediates, and accumulation of ACC and ultimately IR [54–57]. The upregulated acylcarnitine metabolism may play a causal role in insulin resistance. In our study, we confirmed the upregulated acylcarnitine (16 : 0 and 18 : 0) in the T2DM group, which is in line with previous researches [58, 59]. We expect that ACCs could be used to restraint IR and treat T2DM as a potential therapeutic strategy in future.

Frankly speaking, the changes in concentrations of some of the listed lipids may be due to the different diets and drug administration between the two groups, which is an ever-present problem in this field [60], and the exact mechanism still needs more exploration; however, this work provided to us a different view, from which the metabolism of glycerophospholipid and arachidonic acid was associated to diabetes mellitus. These pathways may be essential players in the development of diabetes mellitus, and this view may be helpful in the diagnosis, treatment, and prognosis of diabetes mellitus in future.

## 5. Conclusion

In summary, our study provides a better understanding on recessive progression of diabetes from the perspective of lipids. Lipidomic analysis demonstrated that dyslipidemia associated with T2DM was featured by the boosted levels of FFA (16 : 0), ceramides (34 : 1), and PE (36 : 4) and the reduced levels of lysoPC (24 : 0), PE (36 : 3), and PS (38 : 4). Their significance in pathophysiology was addressed accordingly. Beyond the traditional clinical risk factors, these lipids provided improved assessment of T2DM. Since the particular lipidomics pathway is disrupted, specific lipids could be used as markers for the assessment of severity, course, and prognosis of diabetes, making it possible to implement personalized prophylactic and therapeutic strategies. Nevertheless, in order to better realize its value in individualized treatment, it is warranted to understand the role of these biomarkers in lipid metabolism more deeply. In addition, our study proves that LC-MS-based lipidomics is a powerful approach for the discovery of clinical biomarkers in various diseases.

## Figures and Tables

**Table 1:** ^a^ Clinical information of subjects.

Clinical characteristics	HC (*n* = 47)	DM (*n* = 40)	*p* value
Age (years)	62.60 ± 10.82	64.10 ± 9.42	0.49
Female/male	12/35	12/28	0.82
BMI (kg/m)	25.34 ± 3.22	26.04 ± 2.97	0.29
HCY (*μ*mol/L)	15.28 ± 1.03	14.98 ± 1.09	0.84
Hs-CRP (mg/L)	1.43 (0.66 - 3.90)	1.85 (0.65-5.51)	0.64
HbA1c (%)	5.6 ± 0.38	7.5 ± 1.43	≤0.001
Fasting glucose (mmol/L)	4.98 ± 0.94	6.97 ± 2.19	≤0.001
ALT (U/L)	29 ± 24	24 ± 12	0.22
AST (U/L)	21 (16 - 28)	21 (16 - 26)	0.69
LDH (U/L)	195 ± 90.3	190 ± 85.0	0.80
CK (U/L)	64.5 (52.5 - 92.8)	73.0 (53.5 - 107.0)	0.44
UN (mmol/L)	5.31 ± 1.37	5.70 ± 1.66	0.23
Cr (*μ*mol/L)	78 ± 11	77 ± 21	0.64
UA (*μ*mol/L)	344 ± 92.8	347 ± 78.3	0.87
eGFR (mL/min/1.73 m^2^)	85.12 ± 11.10	86.52 ± 15.50	0.64
HDL-C (mmol/L)	1.08 ± 0.30	0.94 ± 0.22	0.01
LDL-C (mmol/L)	2.19 ± 0.79	2.08 ± 0.78	0.23
TG (mmol/L)	1.53 ± 0.94	1.58 ± 0.69	0.79
TC (mmol/L)	4.03 ± 1.02	3.60 ± 1.08	0.06

^a^Continuous data are presented as mean ± SD or median (interquartile range). HC: healthy controls; DM: diabetes mellitus; BMI: body mass index; HCY: homocysteine; hs-CRP: high sensitivity C-reactive protein; HbA1c: hemoglobin A1c; ALT: alanine aminotransferase; AST: aspartate aminotransferase; LDH: lactate dehydrogenase; CK: creatine kinase; UN: urea nitrogen; Cr: creatinine; UA: uric acid; eGFR: glomerular filtration rate; HDL-C: high-density lipoprotein cholesterol; LDL-C: low-density lipoprotein cholesterol; TG: triglyceride; TC: total cholesterol.

**Table 2:** Significantly changed lipids.

Metabolites	*m/z*	RT (min)	*p* value	Fold change	Change trend
LPC 24 : 0	608.4461	10.5	3.77E-06	0.456	↓
Ceramide 34 : 1	538.5197	14.6	3.68E-06	2.30	↑
PE 36 : 3	740.5366	14.4	2.16E-03	0.417	↓
PE 36 : 4	738.5082	8.8	5.45E-05	3.09	↑
PS 38 : 4	810.5299	13.9	3.08E-04	0.444	↓
FFA 16 : 0	255.2328	6.1	4.54 E-08	3.28	↑

LPC: lysophosphatidylcholine; PE: phosphatidylethanolamine; PS: phosphatidylserine; FFA: free fatty acid.

## Data Availability

Data are available from the authors upon reasonable request.

## Supplementary Materials

Supplementary Materials Supplementary Methods. The experimental and instrumental details. Figure S1: full scan base peak mass chromatograms of QC samples. Table S1: UHPLC gradient for lipidomic analysis.
