# Supplementary material for: LC-MS-Based Lipidomic Analysis of Serum Samples from Patients with Type 2 Diabetes Mellitus (T2DM)
Source: Dis Markers. 2022 Feb 12;2022:5559470. doi: 10.1155/2022/5559470 (PMC8858047; doi:10.1155/2022/5559470)
Supplement: Supplementary Materials — Supplementary Methods. The experimental and instrumental details. Figure S1: full scan base peak mass chromatograms of QC samples. Table S1: UHPLC gradient for lipidomic analysis. [file 5559470.f1.docx]

**LC-MS based lipidomic analysis of serum samples from patients with type 2 diabetes mellitus (T2DM)**

Jia Liu^1, 2^†, Lu Bai^3^†, Weimin Wang^4^, Yuqing Song^3^, Wenbo Zhao^5^, Qingwei Li^5^*, and Qiming Wu^3^*

^1^ Institute of Systems Biomedicine, Department of Pathology, School of Basic Medical Sciences, Beijing Key Laboratory of Tumor Systems Biology, Peking-Tsinghua Center for Life Sciences, Peking University Health Science Center, Beijing, 100191, China

^2^ The First Affiliated Hospital of Hebei North University, Zhangjiakou, Hebei, 075000, China

^3^ Department of Cardiology, Beijing Ditan Hospital, Capital Medical University, Beijing, 100015, China

^4^ Department of Cardiology, Peking University People's Hospital, Beijing, 100044, China

^5^ State Key Laboratory of NBC Protection for Civilian, Beijing, 102205, China

*** Corresponding author contact information**

Qingwei Li

State Key Laboratory of NBC Protection of Civilian

Address: Center Street No.37, Yangfang Town, Changping District, Beijing, China

Phone: 86-10-66758506

Email: [li__qingwei@sina.com](mailto:li__qingwei@sina.com)

Qiming Wu

Department of Cardiology, Beijing Ditan Hospital, Capital Medical University

Address: 8 Jingshun East Street, Chaoyang District, Beijing, China

Phone: 86-10-84322894

E-mail: [13801009058@ccmu.edu.cn](mailto:13801009058@ccmu.edu.cn)

† Jia Liu and Lu Bai contributed equally to this work.

**Supplementary Methods**

**Chemicals and reagents**

Formic acid, HPLC grade methanol, acetonitrile (ACN) and isopropanol (IPA) were obtained from Fisher Scientific. Chloroform was obtained from Tong Guang Fine Chemicals Company (Beijing, China). Ammonium acetate was purchased from Sigma-Aldrich (St. Louis, MO, USA). Ultra-pure water was supplied by a Millipore system (Millipore, Billerica, MA, USA).

**Sample preparation for nontargeted LC-MS analysis**

Lipids and polar metabolites were extracted from plasma samples by a modified Folch method. Specifically, 267 μL CHCl3 and 133 μL methanol were added into 100 μL sample. After vortexing for 10 min, the mixture was centrifuged at 13000 rpm at 4 ℃ for 20 min. The lower organic phase containing lipids was evaporated with a speed vacuum respectively, and the residues were stored at -80 ℃ for further analysis. All samples were processed in the same laboratory to avoid bias.

**Lipidomics**

An Ultimate 3000 ultra high performance liquid chromatography (UHPLC) system coupled to Q-Exactive MS (Thermo Scientific) was used for lipid separation and detection. Samples were reconstituted in 20 μL chloroform/methanol (1:1, V/V) and diluted three times in IPA/ACN/water (2:1:1, V/V/V). After centrifugation at 12000 rpm for 15 min, 5 μL of supernatant were injected for LC-MS/MS analysis.

Chromatographic separation was performed on a reversed phase Xselect CSH C18 column (4.6 mm × 100 mm, 2.5 μm, Waters, USA). Two solvents were used for gradient elution: (A) ACN/water (3:2, V/V), (B) IPA/ACN (9:1, V/V). Both A and B contained 10 mM ammonium acetate and 0.1% formic acid. The gradient program was: 0 min-40% B; 2 min-43% B; 2.1 min-50% B; 12 min 60% B; 12.1-75% B; 18 min-99% B; 19 min-99% B; 20 min-40% B; 25 min-40% B, as shown in Table S1. The column temperature was maintained at 50 ℃, and the flow rate was set to 0.6 ml/min.

Mass spectrometric detection was performed by electrospray ionization in both positive ion mode and negative ion mode. The source voltage was maintained at 3.3 kV in the positive ion mode and 2.8 kV in the negative ion mode. All other interface settings were identical for both positive ion mode and negative ion mode. The capillary temperature, sheath gas flow and auxiliary gas flow were set at 320 ℃, 40 arb and 10 arb, respectively. Data were collected in a data-dependent top 10 scan mode. Survey full-scan MS spectra (mass range m/z 200 to 1200) were acquired with resolution R = 70 000 and AGC target 1e6. MS/MS fragmentation was performed using high-energy c-trap dissociation (HCD) with resolution R = 17 500 and AGC target 1e5. The stepped normalized collision energy (NCE) were set to 15, 30, and 45, respectively. External mass calibration was applied before every sequence run.

The stability of retention time, mass accuracy and intensity is essential in LC-MS based lipidomic analysis. A pooled serum sample was therefore prepared as quality control (QC) to assess the stability of the instrument and ensure the reliability of the data. QC sample was run before, after the sequence, and in every 10 sample runs in the sequence in order to ensure the reproducibility of the data.

**Data processing and analysis**

The acquired raw data were extracted and identified using MSDIAL software according to the instructions in the tutorial. Molecular features not present in 80% of the data were removed from analysis. The accurate mass tolerances of MS and MS/MS were set to 0.01 and 0.05 Da respectively. Statistical significance was calculated using the Student’s t-test, and molecular features were chosen when their p-value was < 0.05. Multivariate analysis was carried out with the MetaboAnalyst 4.0 Web service (http://www.metaboanalyst.ca/). The bar plot in a polar coordinates showing lipids differentially expressed was plotted in R (version 3.4.4).


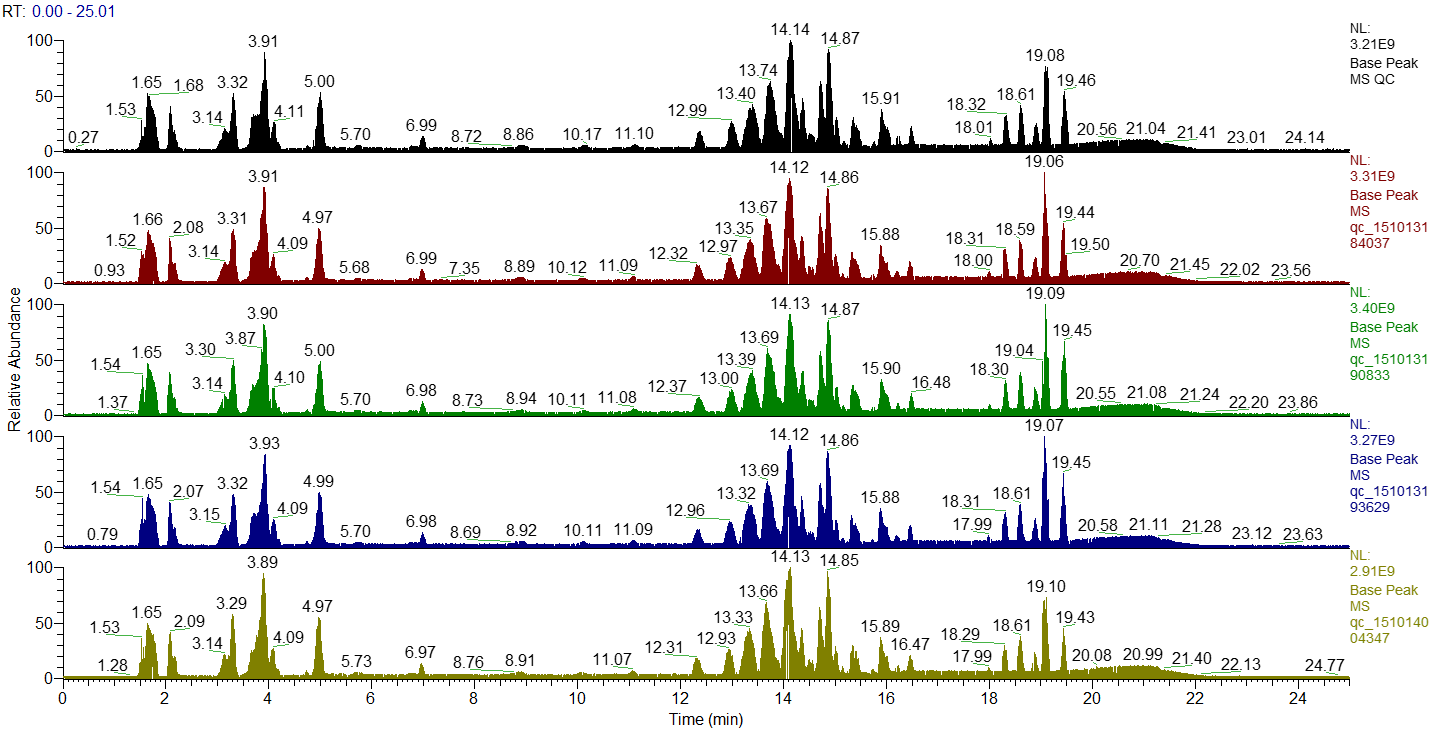


Figure S1. Full scan base peak mass chromatograms of QC samples.

Table S1. UHPLC gradient for lipidomic analysis

Step Time (min) Mobile phase A (%) Mobile phase B (%)

1 0.0 60 40

2 2.0 57 43

3 2.1 50 50

4 12.0 40 60

5 12.1 25 75

6 18.0 1 99

7 19.0 1 99

8 20.0 60 40

9 25.0 60 40
